# Discovering epistasis in large scale genetic association studies by exploiting graphics cards

**DOI:** 10.3389/fgene.2013.00266

**Published:** 2013-12-03

**Authors:** Gary K. Chen, Yunfei Guo

**Affiliations:** ^1^Division of Biostatics, Department of Preventive Medicine, University of Southern CaliforniaLos Angeles, CA, USA; ^2^Zilkha Neurogenetic Institute, University of Southern CaliforniaLos Angeles, CA, USA

**Keywords:** epistasis, GPU programming, CUDA tutorial, high performance computing, gene–gene interactions

## Abstract

Despite the enormous investments made in collecting DNA samples and generating germline variation data across thousands of individuals in modern genome-wide association studies (GWAS), progress has been frustratingly slow in explaining much of the heritability in common disease. Today's paradigm of testing independent hypotheses on each single nucleotide polymorphism (SNP) marker is unlikely to adequately reflect the complex biological processes in disease risk. Alternatively, modeling risk as an ensemble of SNPs that act in concert in a pathway, and/or interact non-additively on log risk for example, may be a more sensible way to approach gene mapping in modern studies. Implementing such analyzes genome-wide can quickly become intractable due to the fact that even modest size SNP panels on modern genotype arrays (500k markers) pose a combinatorial nightmare, require tens of billions of models to be tested for evidence of interaction. In this article, we provide an in-depth analysis of programs that have been developed to explicitly overcome these enormous computational barriers through the use of processors on graphics cards known as Graphics Processing Units (GPU). We include tutorials on GPU technology, which will convey why they are growing in appeal with today's numerical scientists. One obvious advantage is the impressive density of microprocessor cores that are available on only a single GPU. Whereas high end servers feature up to 24 Intel or AMD CPU cores, the latest GPU offerings from nVidia feature over 2600 cores. Each compute node may be outfitted with up to 4 GPU devices. Success on GPUs varies across problems. However, epistasis screens fare well due to the high degree of parallelism exposed in these problems. Papers that we review routinely report GPU speedups of over two orders of magnitude (>100x) over standard CPU implementations.

## 1. Introduction

Large scale population based genome-wide association studies (GWAS) of complex disease have been highly effective at elucidating hereditary risk factors. As of August 1st of 2013, at least 13,841 single nucleotide polymorphisms (SNPs) have been cataloged by NHGRI (Hindorff et al., 2009) as risk variants across 868 diseases and other traits. Despite the apparent success story, reported SNPs account for only a small proportion of heritable variation. For instance, the explained heritability in Type 2 diabetes is approximately 6% even though 18 risk loci have been discovered (Manolio et al., 2009). Given that gene networks are complex, these findings may not be all that surprising. Genes or their regulatory elements are most likely to act in concert via common pathways/networks. Hence it seems reasonable that methods that model SNPs jointly and/or explicitly test for non-additive interactions among them should identify new genetic markers that better explain risk. The computational challenge of exhaustively searching for higher-order interactions, also known as statistical epistasis, is enormous (e.g., a search for pair-wise interactions on the Illumina 1 M Duo microarray would require over $\left(\begin{array}{c} 1e^{6}\\ 2=500 \end{array}\right)$ billion tests). Statistical geneticists have long been interested in developing methods to detect epistasis (Cordell, 2002; Huang et al., 2013). Tackling this challenge requires a multi-prong strategy of advances in statistical methodology, clever optimization algorithms (e.g., those requiring less iterations to converge to a solution), and efficient implementations that extract the full potential of state of the art many-core processors. This review focuses on advances in epistasis research that emphasize the third strategy. We bring attention to some innovative software that scale well to the epistasis problem by harnessing the potential of massively parallel microprocessors on graphics cards, otherwise known as Graphics Processing Units (GPUs). Speedups relative to standard serial implementations of over two orders of magnitude (100 fold) are commonplace. Epistasis detection only scratches the surface of the set of biologically relevant problems which have already been addressed using GPUs, including proteomics (Hussong et al., 2009), phylogenetics (Suchard and Rambaut, 2009; Zhou et al., 2011a), gene-expression analysis (Buckner et al., 2010; Kohlhoff et al., 2011; Magis et al., 2011), high dimensional optimization (Zhou et al., 2010; Chen, 2012), sequence alignment (Campagna et al., 2009; Blom et al., 2011; Vouzis and Sahinidis, 2011; Liu et al., 2012b), systems biology (Liepe et al., 2010; Klingbeil et al., 2011; Vigelius et al., 2011; Zhou et al., 2011b; Liu et al., 2012a), and genotype imputation (Chen et al., 2012). Although there is a rich array of statistical methods designed to search for epistasis, only a few have been developed into code that can make use of GPUs. We encourage readers who are interested in applying or developing state of the art methods to consider implementation strategies that make use of the parallelism in modern processors. Hence, we devote most of this paper's content on basic concepts that are critical for successful implementations. The first section provides a primer on GPU concepts. The following Methods section describes the statistical method and practical issues (such as availability and software prerequisites) behind four programs we successfully tested. This section is also meant to delve deeper on the concepts laid out in the preceding section by presenting vignettes of real life strategies employed by these programs for maximizing performance, each of which is adapted to the underlying statistical method. We provide in the Appendix a training resource for readers with no GPU programming experience who are interested in getting started with minimal effort. This tutorial revolves around a short working example that inclined readers can use to extend toward more interesting problems.

## 2. Primer on creating GPU based algorithms

**Figure 1:**
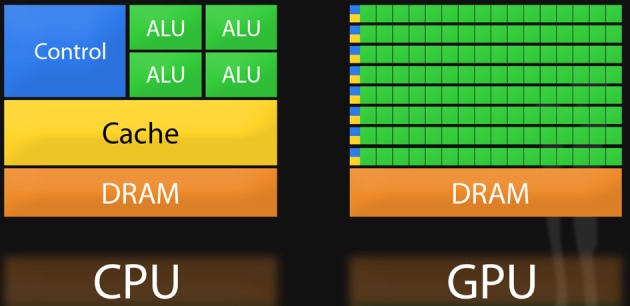
**Comparison of CPU and GPU architecture**.

GPUs are devices installed as adapter cards that slide into any available PCIe (Peripheral Component Interconnect Express) slot, found on modern PC motherboards. The microprocessor on a GPU is known as a SIMD (single instruction multiple data) or stream processors because instructions, such as data loads/stores or arithmetic operations, are simultaneously carried out on several elements of a data stream in a single instruction. Provided that a sufficient number of calculations can be independently carried out in parallel, GPUs can be quite effective in accelerating code. Unlike the CPU, GPU chips are designed with proportionally more transistors dedicated toward floating point logic, as depicted in Figure 1. For this reason, GPUs were originally designed to operate in graphics domains like 3-dimensional rendering and animation since calculations of signal intensities and colors can generally be computed independently over millions of pixels and program logic is homogeneous across pixels. GPU programs have moved into realm of basic science research given the pressing need to develop new ways of analyzing Big Data. New application programming interfaces (APIs) are transforming these specialized devices into workhorses for general problems. GPUs that support such APIs are sometimes called General Purpose GPUs (GPGPUs). GPUs are enabling some previously intractable problems to be completed in days or hours versus years. Searching for epistasis is an example of a problem that maps well to one or more GPUs. This problem is well-structured in that for a given set of input (e.g., all possible pair-wise genotypes for a set of SNPs and an outcome vector), it is easy to keep all available GPU cores busy for virtually the entire time the program is running since calculations for each interaction are “embarrassingly parallel.” The GPU is not a panacea for big computational problems however. For certain classes of problems with less parallelization exposed such that many instructions are required to wait for others to complete before a value can be computed (e.g., some sorting routines), a GPU implementation can be even slower than the serial version of the algorithm. The reason behind this can be understood when comparing CPU and GPU architectures as depicted in Figure 1: in comparison to CPUs, GPUs give up some efficiency in terms of optimization tricks like memory caching and branch prediction (e.g., bypassing “if” statements when possible) for gains in arithmetic throughput.

There are currently many offerings for programming GPGPUs. Many are tailored for programmers who wish to access the power of GPUs with minimal ramp up time. For example, OpenACC (http://www.openacc-standard.org/) allows developers to easily parallize loops written in C, C++, or Fortran code on an “accelerator” such as a multi-core CPU or GPU. The key advantages of OpenACC are easily accessible syntax and portability. The scientific computing toolkit MATLAB is now bundled with support for nVidia GPUs so that programmers can invoke functions on the GPU using familiar MATLAB commands. PyCUDA (http://mathema.tician.de/software/pycuda/) is a wrapper around the CUDA SDK, enabling Python programmers to easily write parallel code. Even statisticians who prefer to work in R can choose among many packages on the CRAN repository, such as *gputools* or *gmatrix*, that interface with any available GPU devices.

However, programmers with highly custom algorithms who need to maximize performance from GPUs will need a greater degree of control over the inner workings of these devices. To this end, two user-friendly APIs are available. In 2006, nVidia Corp announced the release of a proprietary toolkit known as the Compute Unified Device Architecture (CUDA) runtime, targeting a broad audience of C programmers. The toolkit includes an API and a compiler called *nvcc*, which compiles C code containing CUDA extensions into instructions understood by any nVidia CUDA compliant GPU. OpenCL is an alternative open-standard API for GPUs that was originally geared toward a heterogeneous mix of target devices such as cable set top boxes, smart phones, and desktop CPUs. Although OpenCL code is more portable, developing in OpenCL is more labor-intensive than in CUDA. ATI and nVidia, the two sole manufacturers of GPUs, both support OpenCL natively in most of their product line.

When developing code on the GPU, extracting the most speed requires developing a strategy of how data and calculations will be organized. Regarding data, the primary goal is to make use of the high bandwidth (amount of data that can be transferred per time unit) that is unique to GPUs. This property makes parallelization on GPUs more advantageous in many cases than say parallelization over a compute cluster when internode communication is necessary. Memory available to the GPU is categorized at the coarsest level into three levels, where the level with the most abundant memory has the longest latency (i.e., the time it takes to receive data after a request is issued). These three levels can be loosely labeled as system memory, device memory, and on-chip memory. System memory denotes the RAM that is available to the operating system and conventional applications. Device memory, aka global memory, is abundant memory (several GB in many cases) on the GPU that is located on memory banks away from the processing core. On-chip memory is scarce but fast memory adjacent to the GPU computing cores, akin to the L1/L2 caches found on current CPUs. This memory resource is further divided into memory that is visible only to a single thread and that which is shared across threads (threads are explained later in the next paragraph). While semantics are the same between CUDA and OpenCL, they have differing terminology. For example, the on-chip memory resource that is visible across threads is known as shared memory in CUDA while this same resource is called local memory in OpenCL. Performance across these different classes of memory varies greatly. For example, the nVidia Tesla K20 is advertised with a maximum bandwidth of 250 GB/s for memory transfers between global memory and the GPU microprocessors. Bandwidth increases by at least an order of magnitude higher when the cores access shared memory. In stark contrast, in theory only up to 8 GB/s of data can be transferred between system RAM and the GPU because the GPU is an outboard device that interfaces with the host CPU through a PCIe slot. Hence data locality (i.e., the proximity of data to microprocessors) is the greatest determinant of performance. It is imperative then that programs minimize frequent accesses to system RAM, and use device and on-chip memory as much as possible. Because discrete genotypes can be represented as two bits and hence heavily compressed, genome-wide data can be easily accommodated on device memory. Some of the programs we reviewed implement some type of data compression.

**Figure 2:**
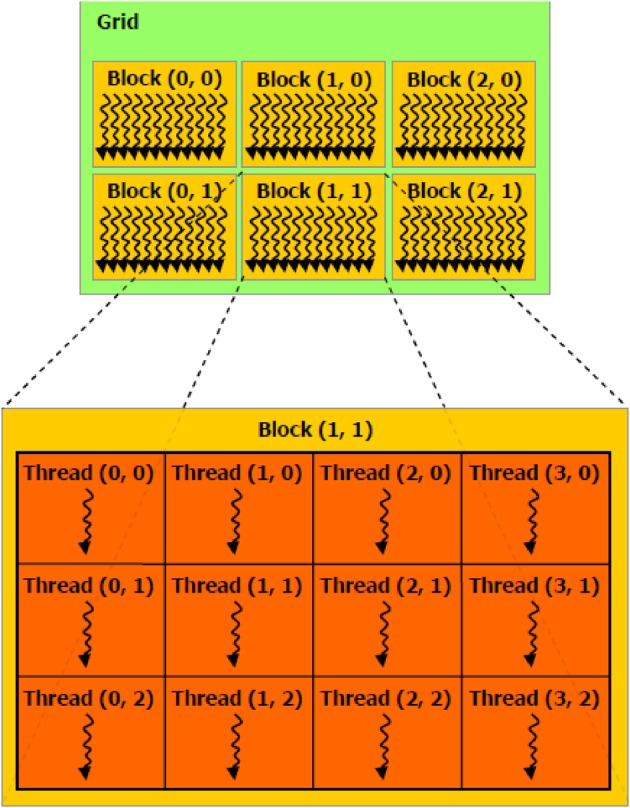
**Parallel execution work-items on a GPU**.

It is also essential to determine how work is parallelized on the GPU. The microprocessors of GPUs process a fixed number of data elements per instruction. On nVidia hardware, this number is known as a warp with value 32 whereas on ATI hardware it is known as a wavefront with value 64. Each element of a data set (or partition of) are mapped into a three-dimensional coordinate layout. These mappings are known as threads and work-items in CUDA and OpenCL terminology, respectively. Threads (or work-items) themselves are grouped into higher-level constructs known as threadblocks (work-groups in the OpenCL spec), which are also mapped in three-dimensions. Figure 2 illustrates this concept in the 2D case where the z dimension is simply sized 1. Since threadblocks can be deployed in any order by the task scheduler on the GPU, the general idea is to group fine-grained but tightly coupled calculations into a threadblocks. In the context of epistasis, one would likely map SNP specific genotypes across a set of subjects into a threadblock. To better understand the semantics of the GPU constructs introduced in this section, Table 1 relates these constructs to the perhaps more familiar entities of a conventional CPU cluster. In the next section we describe four methods implemented on GPUs that put these concepts into practice in the context of epistasis detection.

**Table 1:** **Introduction of GPU constructs using the analogy of a CPU cluster**.

**GPU construct**^a^	**Cluster construct**	**Notes**
Global memory	NAS (network attached storage) array	Plentiful but slow
Shared memory	Local hard drives (temporary scratch space)	Low latency but visible to attached node
Threadblock	Portion of data processed by a specific node	Calculations do not depend on results from other nodes
Thread	Computations parallelizable on a finer scale	Example: using OpenMP to parallelize a for loop

a Terminology as defined in the CUDA specification.

## 3. Methods

Upon searching the literature for epistasis detection methods on GPUs, we were able to locate and download programs for eight published methods, which we list as EpiGPU(Hemani et al., 2011), GLIDE (Kam-Thong et al., 2012), GBOOST(Yung et al., 2011), cuGWAM(Kwon et al., 2012), EPIBLASTER(Kam-Thong et al., 2011a), EpiGPUHSIC(Kam-Thong et al., 2011b), SHEsisEpi(Hu et al., 2010), and MDRGPU(Greene et al., 2010). We evaluate the first four programs of this list, as these were the only ones we were able to successfully deploy on our environment. Table 2 summarizes some key features of these programs. The remaining four programs could not be installed for various reasons. EpiGPUHSIC and EPIBLASTER requires the user to specify a partitioning scheme for large input datasets. Unfortunately, this partitioning logic caused these programs to crash midway through the epistasis screen. We have already notified the author of this bug. SHEsisEpi is software designed to run on a Windows platform so was incompatible in our Linux based environment. Although it is open-source, the code base relies heavily upon Windows libraries. Despite efforts to modify the code, we could not compile it successfully for our environment. MDRGPU requires several Python related libraries to be installed one of which includes PyCUDA (Python wrapper for CUDA). Although we obtained the latest library versions, MDRGPU complained about an issue regarding the PyCUDA version. The authors are working to resolve this issue.

**Table 2:** **Summary of software**.

**Program**	**Trait**^**a**^	**Open source**	**Implementation**	**Availability**
EpiGPU	C	N	OpenCL	http://sourceforge.net/projects/epigpu/
GLIDE	C	Y	CUDA	http://mlcb.is.tuebingen.mpg.de/Forschung/glide/
GBOOST	B	Y	CUDA	http://bioinformatics.ust.hk/BOOST.html#GBOOST
cuGWAM	B	N (linux)	CUDA	http://bibs.snu.ac.kr/cugwam/

a C, continuous; B, binary.

As a prerequisite to running any of the programs featured here, it is necessary that proper GPU drivers are installed and working correctly, which in our case is the proprietary display driver from nVidia (ATI provides a “Catalyst” driver for users with ATI GPUs) and the CUDA runtime. The CUDA runtime includes sample programs that verify a proper installation. In the following subsections, we provide descriptions of each of the four programs. As part of our evaluation, we also designed a basic simulation study and present results to gain further insight into how these programs compare amongst each other.

### 3.1. EpiGPU

EpiGPU is designed to model and test for the association between a continuous trait and a combination of interaction forms (Hemani et al., 2011). Because SNP genotypes are coded with three possible values, there are a total of nine genotype combinations (i.e., interaction forms) that can be constructed for any pair of SNPs. The method performs an 8 d.f. *F*-test to test for significance of a model that includes all interaction forms except the double wild type. For an interaction's genotypes *x* defined using SNP *i* and *j* and a phenotype mean of μ, the following hypotheses are tested:

(1) $$\begin{array}{l} H_{0}:\sum_{i\;=\;1}^{3}\sum_{j\;=\;1}^{3}{\left({\bar{x}}_{ij}-\mu \right)}^{2}=0\\ H_{1}:\sum_{i\;=\;1}^{3}\sum_{j\;=\;1}^{3}{\left({\bar{x}}_{ij}-\mu \right)}^{2}\;>0 \end{array}$$

**Figure 3:**
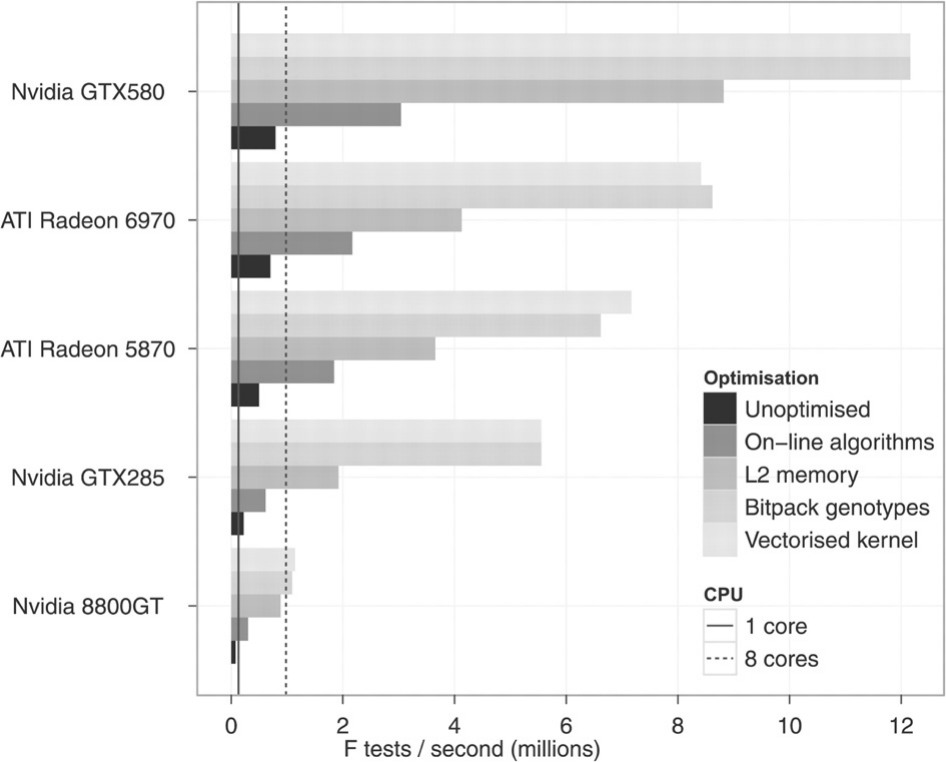
**Improvements from GPU optimizations (figure from Hemani et al., 2011)**.

The program gives the user the option of testing a different set of hypotheses as well, carrying out a potentially more powerful 4 d.f. test. In this test the term *x*_*ij*_ − μ in Equation (1) is replaced with *x*_*ij*_ − *x*_*i*_ − *x*_*j*_ − μ where the middle two terms are marginal means for the genotypes of the two SNPs. The authors employ several optimizations to improve performance, including using on-line algorithms to compute sums of squares and maximizing bandwidth of global memory by compressing genotype data into bitpacked form. Combining different optimization tactics yields improvements of up to 90 fold improvement over a serial version as depicted in Figure 3. Based on the figure, one can see that improving bandwidth makes the greatest impact on performance.

EpiGPU is the only program in this comparison list written in OpenCL; hence it is also the only program that has been evaluated on ATI GPUs. OpenCL programs have one convenient property in that since OpenCL is bundled as part of the GPU driver installation rather than the CUDA runtime installation, users do not need to specify a special search path for shared library objects (e.g., Linux.so files or Windows.dll files) as would be required when running a CUDA based program. The authors distribute EpiGPU as pre-compiled executables, available for the 64 bit Linux, Windows, and Mac platforms. The software is well documented and includes example data sets. Because this program does not require compilation by the user we found EpiGPU to be the easiest program to get up and running.

### 3.2. GLIDE

*GLIDE* is built on the framework of classical least squares multiple regression (Kam-Thong et al., 2012). As such, in contrast to *EpiGPU*, it can support continuous valued inputs such as imputed genotypes, which are often reported as expected dosages. A *p*-value for the interaction (between SNPs *i* and *j*) term is derived from a *t*-statistic with *n* − 4 degrees of freedom. To review, this *t*-statistic is computed as

(2) $$t_{4}ij=\frac{\alpha_{4}^{ij}}{\sqrt{\frac{{\text{Res}}_{\text{SSE}}^{ij}}{m\;-\;4}{\left[{\left(X^{ijT}X^{ij}\right)}^{-1}\right]}_{4,4}}}$$

where the residual sum of squares is

(3) $${\text{Res}}_{\text{SSE}}^{ij}=\sum_{k\;=\;1}^{n}{\left(y_{k}-{\hat{y}}_{k}^{ij}\right)}^{2}$$

and the solution for the interaction term is

(4) $$\alpha_{4}^{ij}={\left(X^{ijT}X^{ij}\right)}^{-1}X^{ijT}y$$

**Figure 4:**
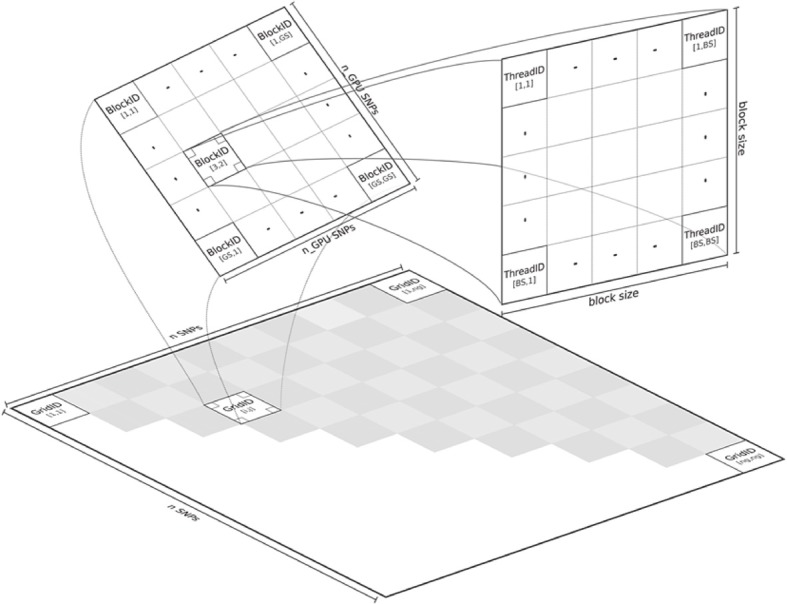
**Parallelization in GLIDE (figure from Kam-Thong et al., 2012)**.

The CUDA based code in GLIDE does a good job of exploiting the high levels of parallelism on the GPU. As shown in Figure 4, the problem of carrying out the full search on $\frac{n(n-1)}{2}$ SNPs is divided at the coarsest level into tiles labeled with “GridID” in the upper shaded triangle. Computations are carried out sequentially across tiles, but in parallel within tiles. If a threadblock dimension of 512 is specified for example, all 512 × 512 interaction pairs of the SNPs assigned to a particular grid are in principle evaluated in parallel; in reality the GPU task scheduler coordinates how many threadblocks can be run at any instance given available resources. A large matrix multiplication operation evaluates *A*^*TA*^ in order to pre-compute correlation matrices, where *A* stores genotypes: columns and rows denoting SNPs and subjects, respectively. The value of *A*^*TA*^ is stored in fast shared memory, so that block specific correlation matrices *X*^*ijT*^ *X*^*ij*^ for any SNP pair *i* and *j* as shown in Equation (4) can simply be extracted from *A*^*TA*^ and re-used in computing Equation (2). In benchmarks against the serial version of PLINK's FastEpistasis option (Purcell et al., 2007), the authors consistently reported speedups of around 2000x over a range of sample sizes.

Source code, documentation, and test data is available and can be compiled on any platform by simply running the make command. GLIDE requires larger datasets to be divided into two input files.

### 3.3. GBOOST

**Figure 5:**
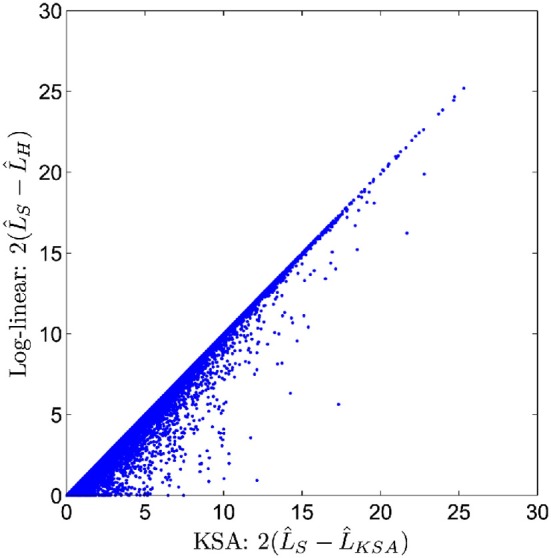
**Candidates from BOOST first stage screen (figure from Wan et al., 2010)**.

**Figure 6:**
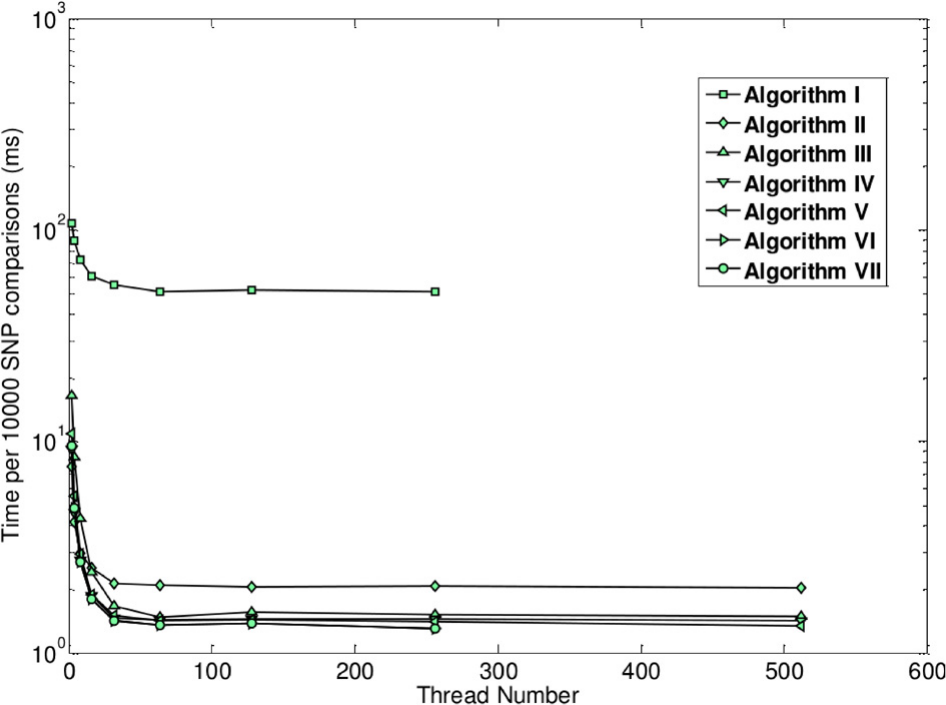
**Runtime as a function of number of active threads across different optimization levels (figure from Yung et al., 2011)**.

Unlike the two previously described methods, the method of GBOOST tests for association between a binary trait and pairwise interactions. Testing for association between any variable and a binary trait is inherently more challenging, since maximum likelihood methods such as logistic regression are too computationally expensive on such a large scale. The strategy used by GBOOST is to apply a set of fast closed form approximations on the entire search space of interactions as described in the paper which first introduced BOOST (Wan et al., 2010). These approximations rule out the vast majority of interactions that show little to no evidence of association to the outcome based on a user specified threshold. Additionally these computations are easier to implement on GPUs than exact solutions. The screening mechanism gains further efficiency by storing input genotypes in a manner that enables fast bit wise operations to be carried out when constructing counts of contingency tables. One needs to be wary of missing true signals when relying upon approximations particular when the user does not apply a liberal threshold. The BOOST method is considered to perform well as a screen since it overestimates the true significance, thus minimizing the false negative rate (Wan et al., 2010). The statistics for the approximation versus the exact solution are shown in the x-axis and y-axis of Figure 5, respectively. Candidates that pass the screen are subsequently tested by computing the log likelihood for a log-linear model using a standard iterative method and comparing this to the log likelihood of the null model. GBOOST is a CUDA implementation of the original BOOST algorithm (Yung et al., 2011). The supplementary material provides an in depth analysis of how different optimization strategies make dramatic impacts on runtime speed. Figure 6 shows run time on the y-axis and the number of threads assigned to each threadblock. Each series in the figure plots performance for a variant of the GBOOST algorithm where higher numbers denote heavier levels of optimization. Not surprisingly, for each algorithm variant, increased parallelism per block improves throughput, reducing run time. The most significant improvement in speed across variants occurs between Algorithm I and II where contingency tables are stored in what's known as constant memory in the former and texture memory in the latter. Constant and texture memory are both cached sections of global memory, but since the GPU architecture allows only a relatively small number of concurrent threads to access constant memory, parallelism can be severely hampered. Algorithm III improves Algorithm II by re-organizing how genotypes are laid out. In particular, by converting the genotype matrix from a SNP major to a subject major ordering data fewer memory fetches from global memory are required, a strategy known as coalesced memory accesses. Diminishing returns are observed with subsequent optimization strategies.

The GPU implementation is approximately 45 times faster than the CPU version. Source code is available and can easily be compiled on all platforms supporting the CUDA runtime through a make file.

### 3.4. cuGWAM

**Figure 7:**
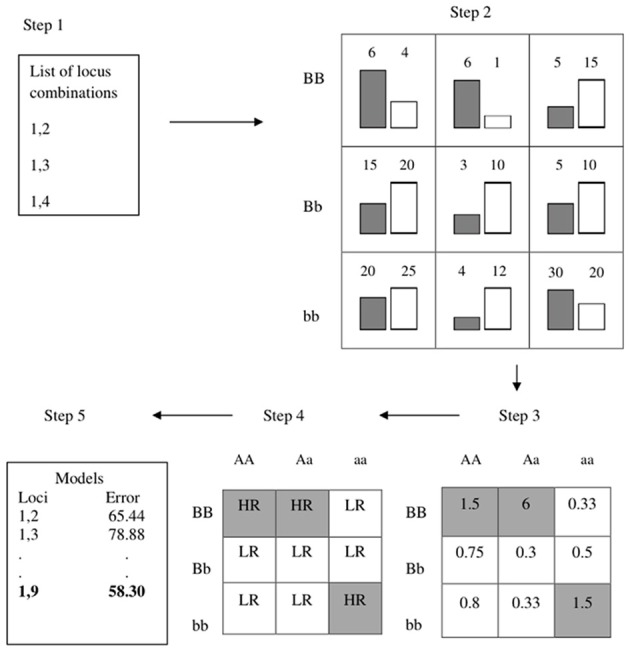
**Overview of MDR (figure from Oki and Motsinger-Reif, 2011)**.

The multifactor dimensionality reduction (MDR) method has been a popular choice for testing for epistasis without strict assumptions about the interaction forms that are implicit in a regression framework for example (Ritchie et al., 2001). Because the method does not rely on any known asymptotics, it employs cross-validation to generate prediction accuracy as a measure of a model's noteworthiness. Figure 7 provides an overview the algorithm: For each pair of SNPs, counts for cases and controls are tabulated at each two-locus genotype for a large proportion of observations known as the training data (e.g., 90%). Based on some threshold, the nine cells are then labeled as high-risk or low-risk. These labels are then used to predict disease status on the remaining “test” observations to evaluate predictive accuracy. The procedure is the repeated for other random divisions of the data to get an averaged predictive accuracy. The computationally demanding nature of cross-validation makes MDR a nice fit for the GPU.

Kwon et al. developed a CUDA implementation of the MDR algorithm called cuGWAM (Kwon et al., 2012). cuGWAM scales well with respect to both sample size and number of markers, with impressive speed ups for the largest problems. For a data set with 2000 markers, speed up increases from 151x with 500 subjects to 652x with 5000 subjects. When the number of markers increases from 500 to 5000 on a dataset with sample size of 2000, speed up rises from 97x to 318x. The authors do not provide specific details as to what strategies they took for optimizing their code other than the fact that they carried out as much of the computations as possible in fast on-chip shared memory.

Running cuGWAM is straightforward. A utility called GMDRconverter is included which converts a MDR format data file into a binary file that can be efficiently read in by cuGWAM. The cuGWAM program allows users to tune GPU performance by specifying the number of threadblocks, threads per block, and the index of the GPU if multiple devices are present.

### 3.5. A direct comparison

After installing the four programs described above on a 64 bit Linux host equipped with two nVidia Tesla K20 GPUs, we initially executed each program using included sample data to verify compatibility. For each program, we recorded average execution time (taken across 100 simulated datasets), and estimated power as a function of false positive rate. As indicated in Table 3 execution times were all similar except for epiGPU, which completed in approximately a tenth of the time required by the other three programs.

**Table 3:** **Average runtime in seconds on simulated data of 10,000 SNPs and 2000 subjects**.

epiGPU	11.36
GLIDE	161.78
cuGWAM	116.63
GBOOST	173.54

**Figure 8:**
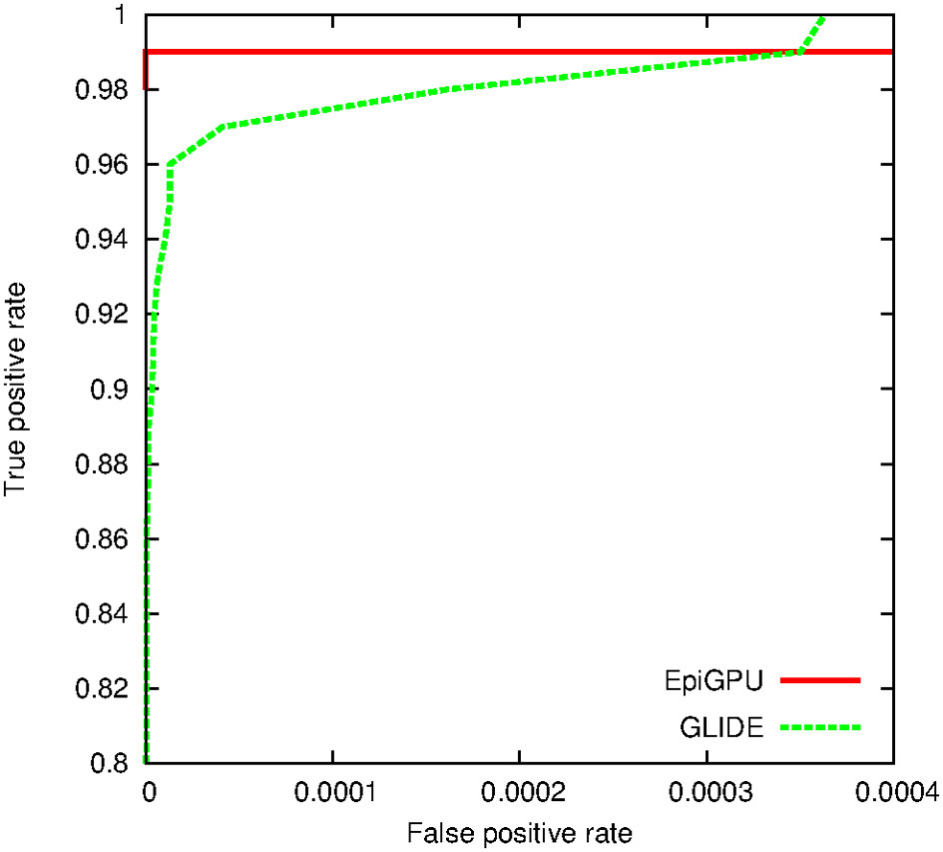
**ROC for methods to analyze a continuous trait**.

**Figure 9:**
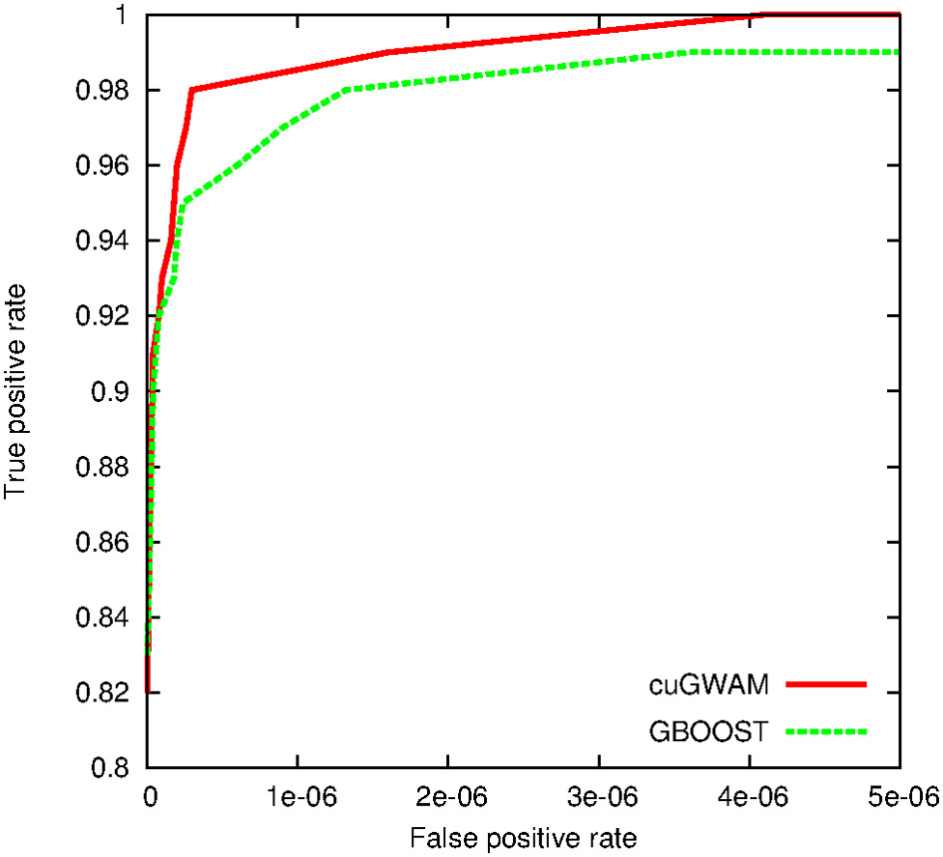
**ROC for methods to analyze a binary trait**.

For the simulation study, we first drew random genotypes on 10,000 SNPs for 2000 subjects assuming linkage equilibrium. A single pair-wise interaction, with an odds ratio of 2.5 and *r*^2^ of 0.6, was then subsequently used to simulate binary and continuous outcomes, respectively. Hundred random distinct datasets were generated for each outcome type. We applied cuGWAM and GBOOST on the datasets with binary outcomes, and epiGPU and GLIDE for datasets with continuous outcomes. The ROC curve shown in Figure 8 compares the power of epiGPU and GLIDE. Both methods display comparable power at this effect size, although epiGPU appears to carry a slight advantage at the lowest false positive rates. However, at looser thresholds (i.e., higher false positive rates), the sensitivity of EpiGPU did not increase, as indicated by the flat curve. For binary traits, Figure 9 plots the comparison of cuGWAM to GBOOST. Interestingly, cuGWAM which is based on the MDR method appears to have slightly more power at this effect size than GBOOST, despite the fact that one might assume a non-parametric method like MDR to have less overall power than parametric methods when analyzing a conventional (e.g., log-additive) interaction as is the case in our simulated data.

## 4. Discussion

In this review we have highlighted four programs designed to efficiently search for epistasis, exploiting graphics hardware traditionally enjoyed by gamers and computer animation specialists. Although the methods behind each of the programs are diverse, our direct comparison indicates that their performance in terms of runtime speed and accuracy are very similar. The program EpiGPU, however, appeared to lead prominently in terms of run time performance compared to its competitor under the category of analyzes using continuous traits, GLIDE. However, GLIDE offers the flexibility of supporting continuously valued genotypes, which could be of value in settings where imputed dosages are the preferred input. Because of limited resources, we did not explore more scenarios such as different number of risk variants, whether main effects also conferred disease risk, effect sizes, and minor allele frequency combinations. One should keep in mind that there may be a greater contrast in power for instance when a disease model includes main effects. Under this context, the 8 d.f. test used in EpiGPU may be more appropriate than the 1 d.f. test for interaction used in GLIDE.

Testing for epistatic models of genetic risk is certainly a research area that has been active and will continue to be as the sources of heritability in common diseases remain elusive. Exhaustive searches for epistasis on even moderately sized datasets (e.g., 500,000 SNPs) are already pushing methods naively parallelized on commodity clusters to their limit. Microprocessor clock speeds can not continue to rise given the density of transistors on today's chips. If we wish to carry out higher dimension searches over other potential genetic predictors such as interactions among SNPs and other molecular phenotypes (e.g., expression levels, methylation status, etc.), we will need to adapt not only by improving statistical optimization methods, but also develop code that also exploits modern many-core processors such as GPUs or multi-core CPUs to extract even more parallelism. We have showcased GPUs as an economical answer to scaling up epistasis software, but we should also remind readers that modern CPUs are beginning to close the performance gap (Lee et al., 2010). For several years Intel CPUs have already provided the market with multi-processors featuring GPU-like properties, a technology known as SSE (streaming SIMD extensions). For example, in C code, programmers can declare SIMD-aware datatypes such as **int4** or **float4**, indicating that four data elements are to be simultaneously processed in one instruction. Advanced compilers such as Intel's MKL (Math Kernel Library) can automatically optimize code further to fully make use of available CPU cores and nearby cache memory banks. The trend is clear that CPU manufacturers are moving to higher core counts. For example, Intel's recent Xeon Phi co-processor sits on a PCI slot like a GPU device, and features up to 61 cores. Barriers of entry are claimed to be lower than those of GPUs, as applications that run on CPUs can be automatically ported to run on these co-processors. It will be interesting to evaluate how these devices perform, particularly for demanding problems that do not fit the GPU programming paradigm well.

### Conflict of interest statement

The authors declare that the research was conducted in the absence of any commercial or financial relationships that could be construed as a potential conflict of interest.

## Figures and Tables

**Figure A1:**
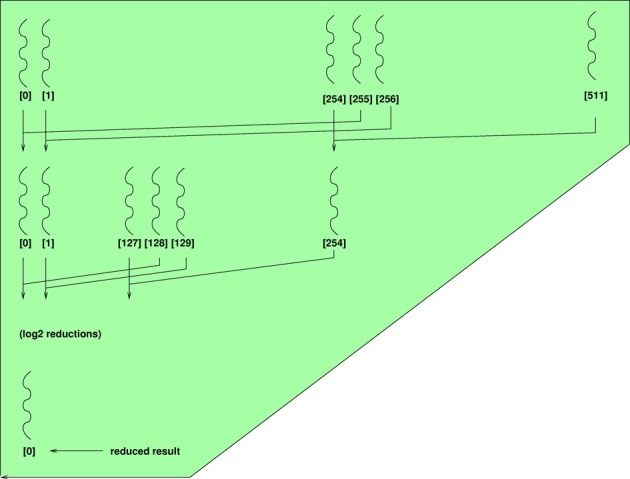
**Parallel reduction for computing summary statistics**.

**Figure A2:**
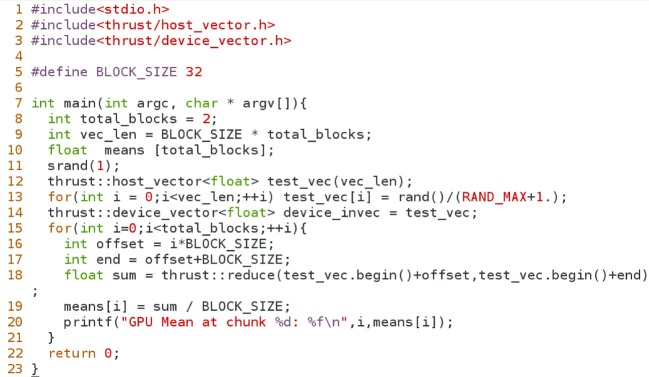
**Thrust code for computing means across different SNPs**.

**Figure A3:**
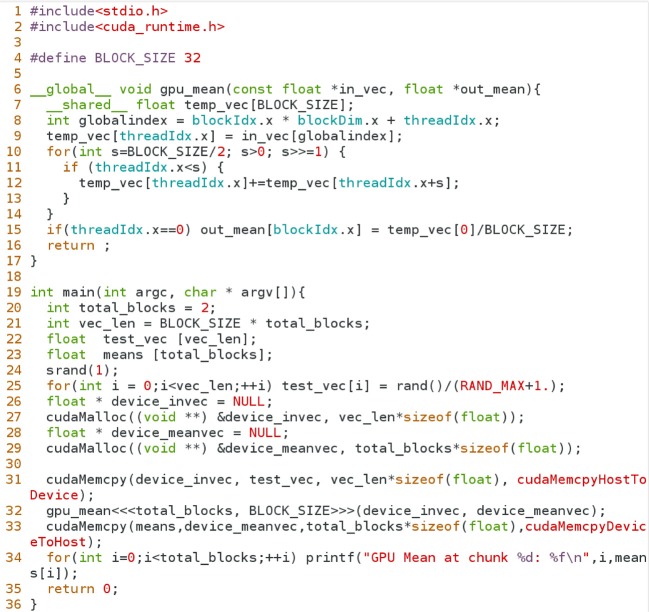
**CUDA code for computing means across different SNPs**.

**Figure A4:**
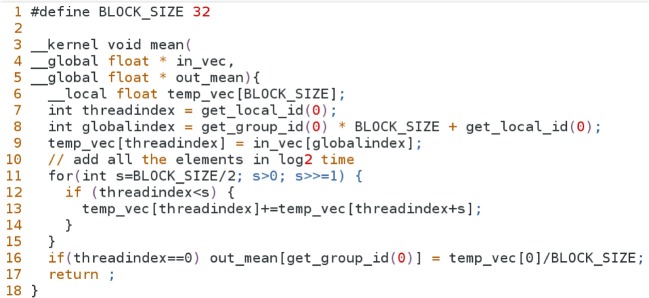
**OpenCL kernel for computing means across different SNPs**.

## 5 Appendix

### 5.1 A hands on tutorial for computing means

In this section we will navigate the reader through the syntax of three development platforms that are bundled in nVidia's toolkit. We will present a simple working example implemented using best practices. The concepts of threads, threadblocks, global, and shared memory should become clearer once readers see them in action.. The goal here is to compute means in parallel on several vectors of floating point numbers, or perhaps in the context of genetics, SNP means.

A commonly applied techniques in parallel programming is known as reduction. Reduction allows a programmer to compute a summary statistic such as the average over a vector of *n* values in less than *n* time steps. This can make quite an impact in problems like epistasis, where means or sums of squares need to be computed on genotypes across millions of interacting predictors. Figure A1 illustrates this concept where each row represents a set of parallel operations that can be completed in a single instruction. At each instruction, a pairwise operation (e.g., addition) is carried between elements of the left and right halves of an array. This recursion continues until one element remains, and requires a total of log_2_(*n*) steps. The technique is easily implemented on GPUs and example code is presented in the next two sub-sections.

#### 5.1.1 Thrust

Thrust is a C++ API that is bundled with the nVidia CUDA SDK. For developers who are familiar with C++ and templates, Thrust features concise, easy to read syntax that abstracts the complexities of writing kernel functions for common tasks such as reductions and sorts.

Let's begin by walking through the 23 line program shown in Figure A2. In this program we plan to store 64 random numbers in an array, but compute the means on the first 32 and the last 32 elements of the array, each mean computed with parallel reductions. Lines 8–11 are ordinary C instructions that set up the array and random seed. In Line 12, we instruct the Thrust run time to set up a vector (array) data structure on the host with the desired length as the parameter argument. The following line populates this host array with random numbers. Line 14 highlights the advantage of Thrust in that it handles the tasks of allocating device memory with the appropriate size and transferring the data to the device in a single concise instruction. Lines 16 and 17 then demarcate the start boundary and one element past the end boundary of the sub-array that we plan to apply the reduction operation on. The Thrust reduction instruction on Line 18 takes in as arguments two C++ style iterators, which in our case point to the indices of the start and end (plus one) elements of the current sub-array.

Notice in this Thrust example we did not need to specify any GPU-specific parameters such as threads, threadblocks, global, or local memory. Thrust manages resource allocations related to these constructs internally. The drawback, however, is a loss of control for more customized algorithms. As with any API, this is the price one pays for more abstraction. Suppose we wanted both reductions to be carried out in a single kernel call (and performed in parallel given sufficient cores). In the following two examples, we will present the equivalent problem using CUDA and OpenCL that allows the programmer to explicitly encode such a design.

#### 5.1.2 CUDA

In this section we will walk through a minimal CUDA program (shown in Figure A3) that computes genotype means. It is recommended that programmers handle catch errors/exceptions that are returned by the CUDA runtime, but for the sake of focusing on core concepts, we have removed such error handling code from this example. To simplify this example further, we limit vector length to 32, which is the size of an nVidia warp (the minimum number of data elements that can be read in a single transaction). For longer vector lengths we would need to introduce the concept of thread barriers, a slightly more advanced concept that would enable more parallelism but is not necessary for a minimal implementation.

We begin in lines 20–25 of the program, initializing variables on the host using standard C syntax. From lines 26–29 we perform a set of instructions that allocate two memory buffers on the GPU: one for the set of input data arrays (stacked into a single array), and the other for the output array containing the computed means. Line 31 instructs the CUDA runtime to transfer data from the host arrays to the memory buffers so that these data will be accessible by the GPU microprocessors. The following line is the actual invocation of the function that is to run on the GPU device, where parameters are enclosed by triple angle brackets. Line 33 is analogous to Line 31 and moves data back to the host, in this case the computed means.

The section defined by lines 6–17 is the heart of the program, which contains the set of instructions that are executed by the GPU microprocessors. Functions that are run on the GPU are qualified with either the string **__global__** or **__local__**, where only the former qualifier can be invoked by the host program. In line 7, we declare an array of floating point numbers to store genotypes for a particular SNP. This array is scoped with keyword **__shared__** to specify that the data is to be stored in the fast memory bank near the computing cores. This is vital to avoid frequent accesses to slow global memory (such as the **in_vec** and **out_mean** variables in our example). We organize our problem so that threadblocks are on a one-dimensional grid, each threadblock mapping to a SNP. Furthermore, threads within a threadblock map to the individual genotypes of a SNP. Hence, line 8 computes the proper index of the element in **in_vec** that is required for copying genotypes from global memory into shared memory, as shown in line 9. The recursive reduction procedure described above is carried out in the for loop in lines 10–14. Finally, since we only need to report one value (the mean), line 15 instructs the first thread to divide the result of the sum reduction by the number of genotypes, and store the result in the global variable **out_mean**.

#### 5.1.3 OpenCL

Here we present the same algorithm for computing genotype means, implemented in OpenCL. One major difference in OpenCL programs is that code that runs on the GPU (known as kernel code) is always contained in a file that is separate from the host code, whereas in CUDA a programmer can choose to organize the source code in either fashion. Because OpenCL programs are structured as such, host code require substantially more lines of code, as the kernel source must be read in from a file, and compiled at run time. The supplementary document provides a listing of the OpenCL implementation of host code that invokes our example kernel. Comparing Figure A4 to the kernel function in Figure A3, one immediately notices that there are only subtle differences in syntax between the two platforms. Notable differences include the use of keyword **__kernel** that replaces the CUDA keyword **__global__**, and the qualifier **__global** for variables **in_vec** and **out_mean** (CUDA defaults to global memory scope when no qualifiers are present). Fast **__shared__** memory is scoped as **__local** in OpenCL. We obtain the threadblock ID and thread ID using the functions *get_group_id()* and *get_local_id()*, respectively.
